# Applying convolutional neural networks to speed up environmental DNA annotation in a highly diverse ecosystem

**DOI:** 10.1038/s41598-022-13412-w

**Published:** 2022-06-17

**Authors:** Benjamin Flück, Laëtitia Mathon, Stéphanie Manel, Alice Valentini, Tony Dejean, Camille Albouy, David Mouillot, Wilfried Thuiller, Jérôme Murienne, Sébastien Brosse, Loïc Pellissier

**Affiliations:** 1 Department of Environmental System Science, ETH Zürich, 8092 Zurich, Switzerland; 2 Swiss Federal Research Institute WSL, 8903 Birmensdorf, Switzerland; 3 CEFE, Univ. Montpellier, CNRS, EPHE-PSL University, IRD, Montpellier, France; 4 SPYGEN, Le Bourget-du-Lac, France; 5 DECOD (Ecosystem Dynamics and Sustainability), IFREMER, INRAE, Institut Agro - Agrocampus Ouest, Rue de l’Ile d’Yeu, BP21105, 44311 Nantes Cedex 3, France; 6 MARBEC, Univ. Montpellier,CNRS, IRD, Ifremer, Montpellier, France; 7 Institut Universitaire de France, IUF, 75231 Paris, France; 8 CNRS, LECA, Laboratoire d’Écologie Alpine, Univ. Grenoble Alpes, Univ. Savoie Mont Blanc, 38000 Grenoble, France; 9 Laboratoire Evolution et Diversité Biologique (UMR5174), CNRS, IRD, Université Paul Sabatier, Toulouse, France

**Keywords:** Classification and taxonomy, Biodiversity, Biodiversity

## Abstract

High-throughput DNA sequencing is becoming an increasingly important tool to monitor and better understand biodiversity responses to environmental changes in a standardized and reproducible way. Environmental DNA (eDNA) from organisms can be captured in ecosystem samples and sequenced using metabarcoding, but processing large volumes of eDNA data and annotating sequences to recognized taxa remains computationally expensive. Speed and accuracy are two major bottlenecks in this critical step. Here, we evaluated the ability of convolutional neural networks (CNNs) to process short eDNA sequences and associate them with taxonomic labels. Using a unique eDNA data set collected in highly diverse Tropical South America, we compared the speed and accuracy of CNNs with that of a well-known bioinformatic pipeline (OBITools) in processing a small region (60 bp) of the 12S ribosomal DNA targeting freshwater fishes. We found that the taxonomic labels from the CNNs were comparable to those from OBITools, with high correlation levels for the composition of the regional fish fauna. The CNNs enabled the processing of raw fastq files at a rate of approximately 1 million sequences per minute, which was about 150 times faster than with OBITools. Given the good performance of CNNs in the highly diverse ecosystem considered here, the development of more elaborate CNNs promises fast deployment for future biodiversity inventories using eDNA.

## Introduction

Effective ecosystem governance and management require an increase in speed, accuracy and ease of collecting and processing of biodiversity data^31,49^. Biodiversity data collection requires a shift in focus from expert monitoring towards high-throughput data acquisition technology^24^. Conventional biodiversity monitoring approaches are labor intensive, depend on expert knowledge—resulting in long delays between sampling and results^53^, and miss many species that are either small, rare, cryptic or elusive^41^, which in turn hinders accurate ecological interpretations. Fortunately, our ability to rapidly generate inventories of whole species communities is improving with the emergence of environmental genomics, specifically environmental DNA (eDNA)^6,25,27,75^. All organisms living in an ecosystem shed tissue material, which can be detected through eDNA metabarcoding^74^, offering an integrative view of the ecosystem composition^27,33^. Coupled with high-throughput DNA sequencing methods, eDNA metabarcoding can help with the rapid assessment and monitoring of biodiversity across all levels of life, from prokaryotes to eukaryotes^40^, with a higher detection capacity and cost-effectiveness than traditional methods^59^. The reads from high-throughput amplicon sequencing of eDNA can be compared with reference barcode libraries, enabling the establishment of taxonomic lists directly from environment samples^74^. Ultimately, these lists can be used to assess ecosystem functioning and health status^25^. With an increasing number of initiatives proposing the use of eDNA metabarcoding routinely and globally to monitor ecosystems^5^, the processing of such massive sequencing data will require novel automated bioinformatic solutions that are both fast and accurate.

As the laboratory molecular steps of eDNA metabarcoding have gained in efficiency^69,75^, the major bottleneck and technical challenge has shifted from the development of efficient sampling and laboratory protocols to the processing of the produced large set of raw sequencing data into taxonomic lists^32^. In particular, eDNA metabarcoding amplifies small DNA sequences (‘barcodes’), typically 60–300 bp long, from the mitochondrial genome for use with Illumina sequencing technology^71^. This sequencing process generates a huge quantity of small sequence reads that require fast and accurate bioinformatic processing to be interpreted^34,35^. The bioinformatic processing includes several steps (merging the forward and reverse reads, demultiplexing, dereplicating, filtering by quality, removing errors), after which the retained and cleaned sequences are assigned to a taxonomic label^32,50,56^. Taxonomic labelling then involves transforming sequence reads from eDNA into lists of taxa that can be used by experts and scientists to understand biodiversity patterns, structures and dynamics of assemblages. They can additionally be used for management decisions^68^, based on the detection of rare^9,63^, endangered^37^, or invasive species^68^. Given that most existing pipelines are time consuming to apply^52^, efficient algorithms transforming eDNA reads into accurate taxonomic lists using machine learning could potentially enable efficient and parallel automatization on cloud infrastructure for a broad application of eDNA technology^65^.

Compared with traditional bioinformatic approaches^52^, machine learning could increase the efficiency and capacity of the taxonomic labelling of eDNA reads^55^. Deep learning has revolutionized object classifications in various biological applications, from identifying species on images^38^ to modelling species distributions in habitats^28^. Taxonomic groups represent discrete classes that can be related to sequence features, including the composition and distribution of nucleobases within DNA sequences^14,39^. For example, k-mer summarizes the counts of nucleotides within sub-sequences of length k and, in combination with machine classifications, have been used to label sequences from bacteria, archaea, fungi and viruses^58^. The association between k-mer features and taxonomic labelling can be trained in a neural network from a reference genetic database^58,60^ to predict the label of any new sequence. Alternatively, a convolutional neural network (CNN) can self-learn a broader range of spatially organized DNA base-motif features existing in the DNA sequences^39^. The neural structure subsets signals from a restricted region of the input data known as the receptive field and responds to localized patterns in the sequence data. The numeric encoding of the four DNA bases makes it possible for the spatial placements of nucleotides to be interpreted by the CNN. In particular, Busia et al. developed a CNN^14^ which trains a deep neural network to predict database-derived taxonomic labels directly from query sequences. Hence, preliminary use of machine learning with DNA sequence data shows the potential of this approach for taxonomic labelling^14,44^, but so far it has mainly been used to label relatively long amplicons such as the full 16S gene, in fragments up to 250 bp long^14^. It remains to be determined how it performs in the taxonomic labelling of short sequences from eDNA metabarcoding.

The most computationally costly step in the processing of eDNA metabarcoding is data cleaning^52^, and a large computational gain from machine learning could be achieved if a CNN can be applied directly on raw sequencing data that can contain many errors, including PCR substitutions or insertions or deletions of bases^66,67,73^. Existing eDNA bioinformatic pipelines apply a computationally demanding process of sequence processing and cleaning^52^, conserving only high-quality reads^10^ before the taxonomic labelling of DNA reads. To circumvent this data cleaning procedure, CNNs should be able to either identify low-quality sequences or accommodate noisy data in the taxonomic labelling. CNNs with data augmentation have been used to render networks more robust to noisy data, for example by adding random variation in the training data^70^. Busia et al.^14^ artificially introduced variation into sequences within the reference database to build a more robust CNN, adding between 0.5 and 16% of mutations by switching DNA bases randomly^14^. While the authors found that moderate artificial noise rendered the network more robust to potential sequencing errors, setting an excessive value decreased the CNN performance. Furthermore, the CNN should be trained to tolerate the library tags and the PCR primers present in raw metabarcoding data, but these aspects have remained largely unexplored. The CNN could then be used to process and identify the sequences from raw metabarcoding files, independently of the processing step in which they are demultiplexed to each sample. If reliable, a CNN pipeline serves as a revolutionary tool to process the exponentially growing quantity of eDNA metabarcoding data used to characterize ecosystems.

**Figure 1:**
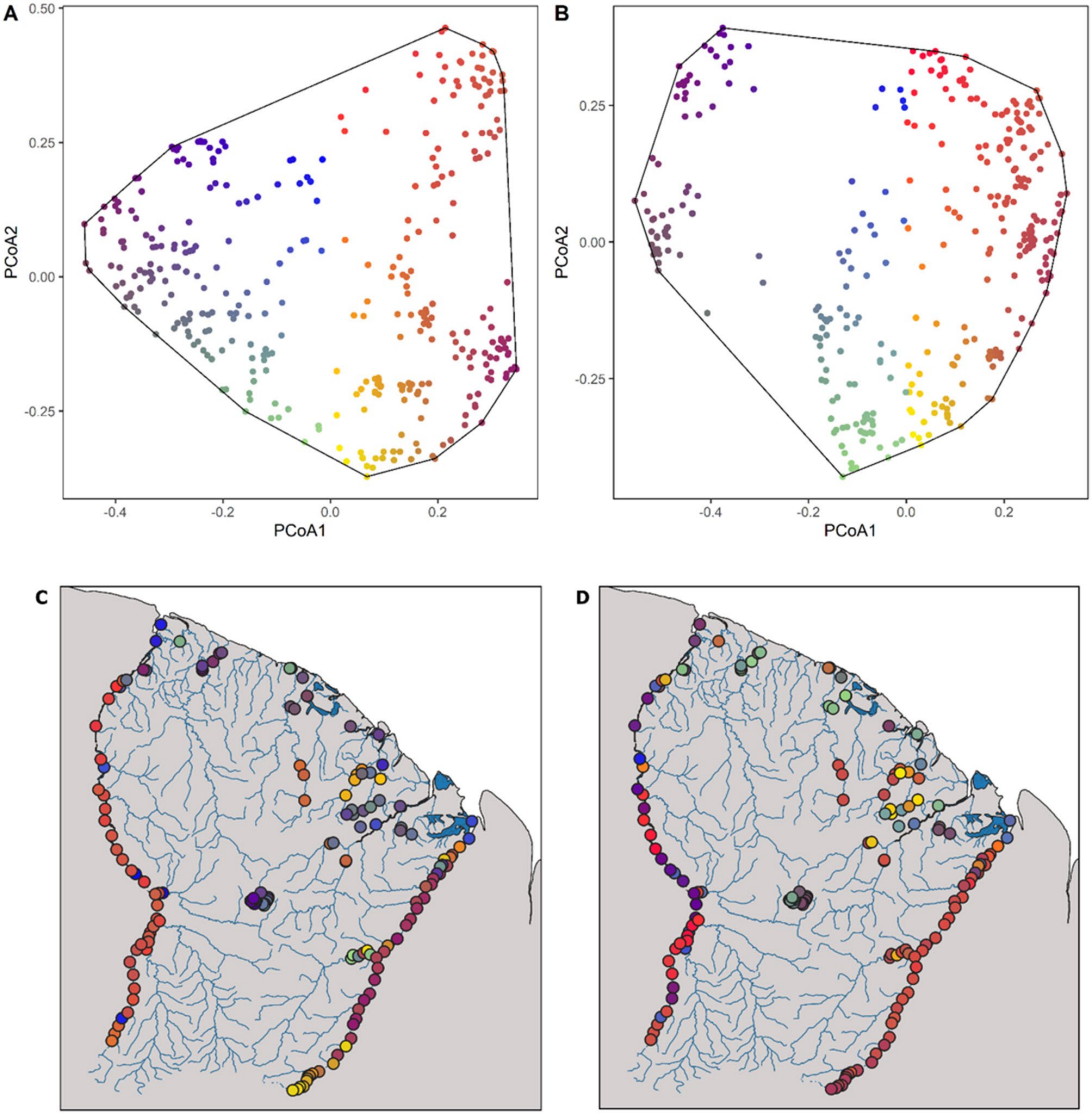
Principal coordinate analysis (PCoA) of species composition dissimilarity between filters. (**A**) Ordination of filter species composition dissimilarity in the outputs of OBITools. (**B**) Ordination of filter species composition dissimilarity in the outputs of the CNN applied to raw reads. Dissimilarity matrices were built with Bray–Curtis distances on read abundance per species per filter. (**C**) Maps of the filter locations, coloured according to the position of the filters in the PCoA space for OBITools outputs. (**D**) Maps of the filter locations, coloured according to the position of the filters in the PCoA space for the CNN applied to raw reads outputs. The maps were created with QGIS 3.6.1.

Here, we used a comprehensive eDNA data set collected in tropical South America to evaluate the ability of CNNs to rapidly and accurately process eDNA metabarcoding files into taxonomic labels. We built CNNs that allow the processing of short sequences produced by eDNA metabarcoding and tested whether the accuracy and speed of CNNs are comparable to those of OBITools^10^, a widely used pipeline to process eDNA data. As a case study we used one of the largest standardized eDNA data sets currently available for fishes, corresponding to a multi-year campaign effort to sample the tropical South American rivers of French Guiana^54^ (Fig. 1). This eDNA data set is associated with a quasi-exhaustive reference database covering most of the known species of the region for the ‘teleo’ region of the 12S rRNA mitochondrial gene^22,26^. The raw data set contains nearly 700 million sequences, with about 205 million sequences belong to the samples of interest here. The freshwater ecosystems of French Guiana are among the most species-rich ecosystems for riverine fishes globally^3^, and among the rivers the least impacted by humans^72^. Good performance of an approach in a complex ecosystem provides a robust proof of concept for further applications in any other ecosystem with a simpler species assemblage. Within this general processing framework and using this case study, we asked the following questions: (1) How does a CNN approach perform in the training of eDNA sequence classification for labels of the reference database? (2) How robust is the classification of a CNN applied directly to raw Illumina metabarcoding short sequences? (3) How do a classical metabarcoding pipeline and our CNN approach compare with the pre-existing information about biodiversity composition within two river catchments with a long history of traditional sampling?

## Results

### CNN training and evaluation with split sampling

CNNs learned features of the 60 bp teleo sequence reads with good internal and external predictive power. Larger networks did not necessarily produce better results, indicating low overfitting. A CNN of moderate complexity learned the full structure contained in the training sequence data. The training and evaluation of the CNN with split sampling considered 156 species (out of 368) which had at least two unique sequences. The optimal CNN consisted of a 150 $\times$ 4 unit input layer, one convolutional layer of 4 filters with a 7 $\times$ 4 extent, 3 dense layers with 128 neurons each, and an output layer 156 neurons wide. On the training data, the networks achieved 92% accuracy, with small differences between the networks trained on the base reference data and those trained on the augmented reference data (i.e. with added tags, primers and reverse complements). When applying the CNN to the hold-out data (316 sequences from the 156 species), we found an accuracy of 91% on the base data and 89% on the augmented data. When an optimized 0.9 binarization threshold was used with the F-beta metric, the accuracy rose to 98% for both CNNs, at the cost of  16–26% of the predictions being discarded for the base and augmented data, respectively. We then used the entire data set in the training process, using all 368 species, and repeated the analyses for the base and augmented data. The optimal CNN was similar to the previously chosen networks, with a single convolutional layer of 4 filters with a 7 $\times$ 4 extent, followed by 2 dense layers each 384 neurons wide. With these networks, training accuracy was similar to that from the split evaluation at 92%. Validating the networks on the reference sequences yielded higher accuracies of 96% and 94% for the base and augmented CNNs, respectively. With a binarization threshold of 0.9, the accuracy rose to 99% for both the base and augmented data sets, at the cost of rejecting 9–13% of all sequences evaluated (Supplementary Material Fig. 1). We used a binarization threshold of 0.9 for all further evaluations.

### CNN application on the raw and cleaned eDNA data set

**Figure 2:**
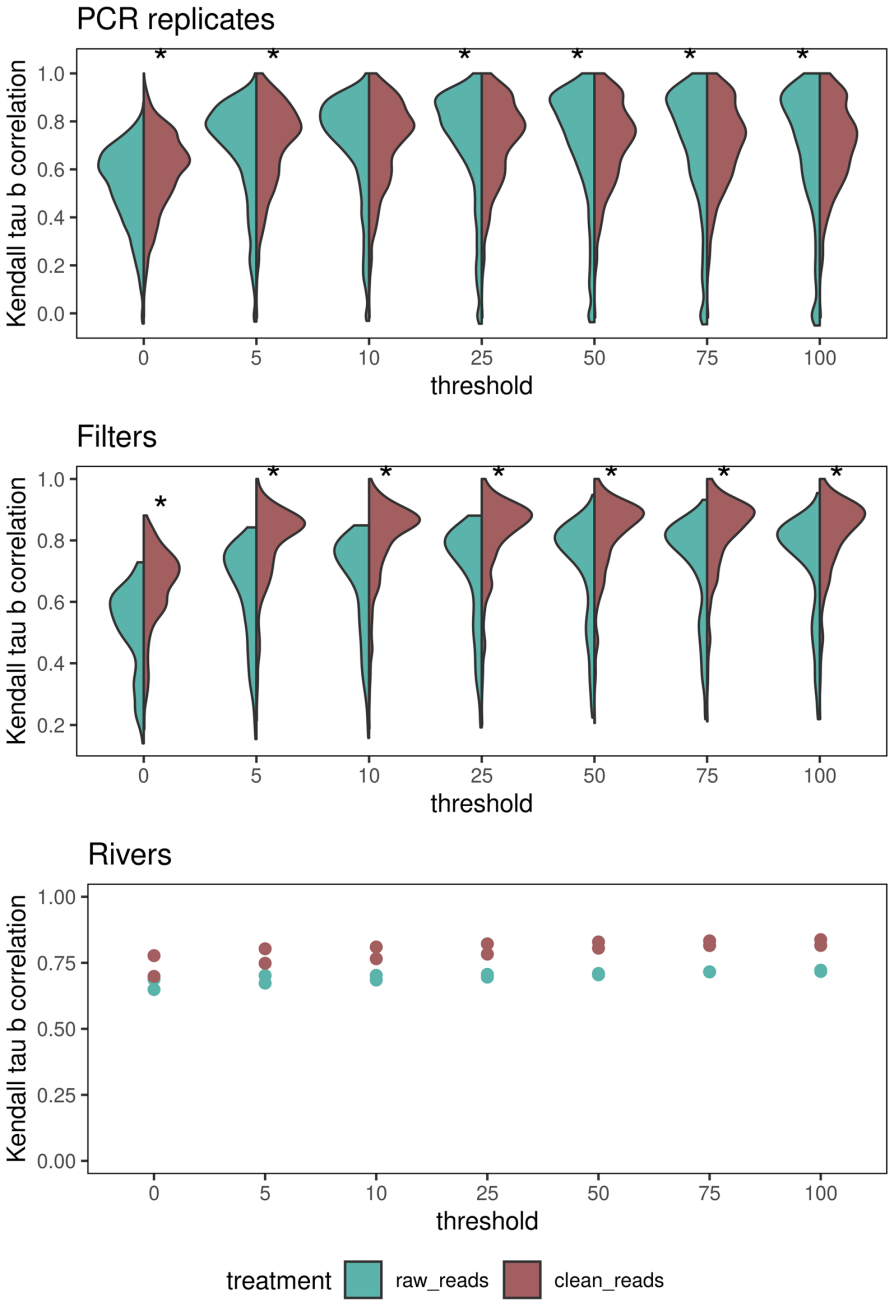
Kendall Tau-b correlation coefficient between the outputs of the CNN and OBITools. The left side of the violin plots (blue) displays correlation values between OBITools and the CNN applied to raw reads. The right side of the violin plots (red) displays correlation values between OBITools and the CNN applied to clean reads. The x-axis represents the threshold of the minimum read number per species for the species to be considered present. Stars represent a significant difference between the two correlations. The analysis was made at three levels: PCR replicates (top), eDNA filters (middle), and rivers (bottom).

**Figure 3:**
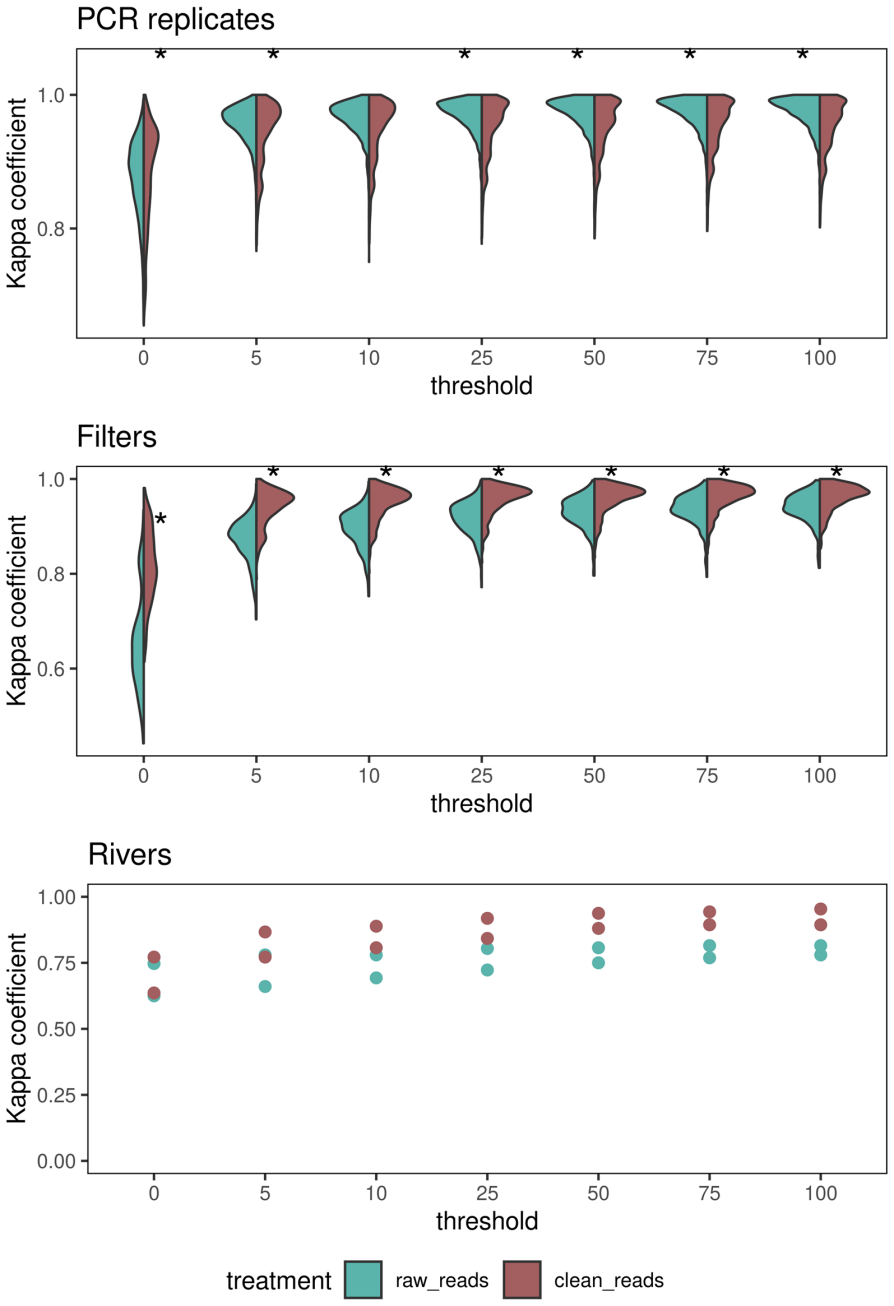
Kappa correlation coefficient between the outputs of the CNN and OBITools. The left side of the violin plots (blue) displays correlation values between OBITools and the CNN applied to raw reads. The right side of the violin plots (red) displays correlation values between OBITools and the CNN applied to clean reads. The x-axis represents the threshold of the minimum read number per species for the species to be considered present. Stars represent a significant difference between the two correlations.

We found that there were limited differences in the output between the CNNs trained on the raw sequence data compared to those trained on cleaned data. To attenuate sequencing noise in the analysis, we considered a second threshold of the minimum number of reads required for a species to be retained. We compared the number of reads per species needed for each CNN with that needed for OBITools and observed that the median Kendall Tau-b correlation increased when a more stringent threshold on the minimum number of reads per species was applied to all levels of sample aggregation. An optimal threshold of 50 reads per species resulted a slightly better correlation for clean (median Kendall Tau-b = 0.77, range 0.22–0.94) than for raw reads (median Kendall Tau-b = 0.84, range = 0.2–1, Fig. 2) at the filter level. The same effect persisted on the PCR replicate and river levels. We considered only species with more than 50 reads within a PCR replicate in the following analyses. We repeated the analysis using the kappa similarity measurement (Fig. 3). The CNN applied to the clean reads (after assembling and demultiplexing) had a slightly higher composition similarity (median kappa value 0.96, range 0.83–1.0) than that applied to the raw reads directly from the Illumina outputs (median kappa value 0.93, range 0.79–0.99). The kappa values are based on the predicted presence and absence of species. Hence, the results were slightly better than those from the Kendall Tau b values, as those take the relative abundance of the predictions into account. All approaches recovered similar gradients of composition, differentiating between coastal and upstream assemblages (Fig. 1). The composition difference between methods resulted from a slightly larger number of species predicted by the CNN (median species number 63) than by OBITools (median species number 56). Furthermore, the CNNs still lacked feature parity with OBITools with regard to ambiguous sequences, which can result in more pronounced differences in the OBITools output.

### Validation with the known species list of the region

**Figure 4:**
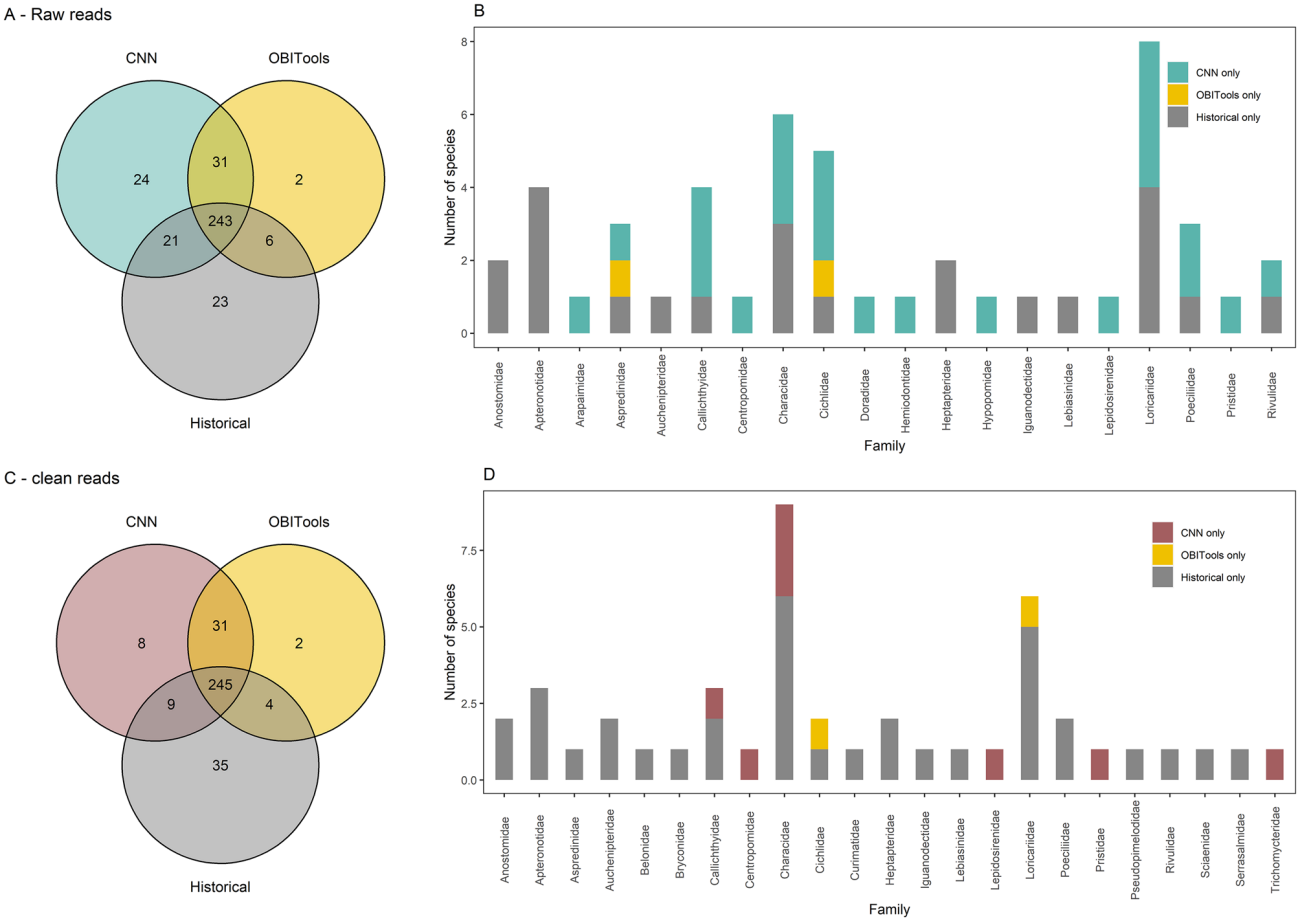
Species detections with the CNN approach, with OBITools, and in historical records in the combined Maroni and Oyapock rivers. (**A**) Overlap of species detections between the CNN applied to raw reads (blue), OBITools (yellow) and historical records (grey). (**B**) Number of species per family, detected with only one method (CNN applied to raw reads, OBITools or historical records). (**C**) Overlap of species detections between the CNN applied to clean reads (red), OBITools (yellow) and historical records (grey). (**D**) Number of species per family that were detected with only one method (CNN applied to clean reads, OBITools or historical records).

We found a major overlap between historical records and the species composition recovered from the CNN. The data synthesis across historical fish surveys yielded a total of 351 species in the Maroni and Oyapock rivers, 293 of which were present in the reference database and thus potentially detectable with eDNA. For both rivers combined, the CNN applied to raw reads assigned 319 species, 264 of which were known from the historical records, while 55 had never been recorded before (Fig. 4a). The CNN and OBITools detected 274 species in common, while the CNN retrieved 21 species known from the historical surveys in these rivers that were not retrieved with OBITools but identified 24 species not known from the survey synthesis or identified with OBITools. The species detected only with the CNN mainly belong to the Loricariidae, Cichlidae, Characidae and Callichthyidae families. The 23 species known from historical records and not detected by either eDNA method mainly belong to the Loricariidae, Characidae, Apteronotidae and Anostomidae families. The two species detected only with OBItools are from the Cichlidae and Aspredinidae families (Fig. 4b). The CNN applied to clean reads detected 293 species, 254 of which were present in the Maroni and Oyapock synthesis, 276 of which were also found in the outputs of OBITools, 9 of which were found only with the CNN and in the synthesis, and 8 of which were found only with the CNN. In the case of OBITools, 282 species were detected, 249 of which were included in the historical synthesis and 33 of which had never been recorded in the Maroni or Oyapock rivers (Fig. 4c). The species detected only with the CNN mainly belong to the Characidae family. The species known from historical records but not detected with either eDNA method belong to the Loricariidae, Characidae and Apteronotidae families. The two species detected only with OBITools are from the Loricariidae and Cichlidae families (Fig. 4d). The same analysis at the single river scale provided similar results (Supplementary Material Figs 4, 5). Hence, while both methods detected species not found in the historical records, the CNN generally recovered more species than OBITools, which could correspond to either new true observations or commission errors. The CNN applied to raw reads retrieved more species that were not in historical records nor found with OBITools. For the Maroni river, the CNN applied to raw reads and the CNN applied to clean reads retrieved 232 species in common, while 48 were found only with the raw reads and 16 only with the clean reads. For the Oyapock river, 185, 66 and 18 species were found in common, only with the raw reads, and only with the clean reads, respectively (Supplementary Material Fig. 6).

### Computation time

Overall, the CNN processed approximately 1 million input sequences per minute, compared with 20,000 input sequences per minute for OBITools. For the CNN, we distinguished between two computational efforts, which were measured independently: (1) network training, which needed to be performed once per reference database, and (2) the application on field data. Training a network on the augmented and complete reference database currently took around 10 min on an Nvidia Titan RTX GPU. Training a network on the clean reference database was faster and takes  6 min on the same GPU. The training and application time is dependent on the size of the input data and the network size. A large part of the computational time for the OBITools pipeline is dedicated to the alignment (up to  80%) and demultiplexing (up to  15%) steps. By training and applying a convolutional neural network directly on raw reads, we could sidestep this issue completely and achieve significantly faster processing times and lower power consumption at the cost of more marked differences in the recovered compositions overall.

## Discussion

The monitoring of biodiversity in highly species-rich ecosystems has generally been challenging, with gaps in biodiversity data existing in the tropics^23^. eDNA metabarcoding is a revolutionary method that can enhance the monitoring of species in complex ecosystems^48^, but is associated with the challenge of rapidly processing large data sets. Our study demonstrates the application of a CNN to process short eDNA sequence reads directly from raw sequencing Illumina outputs. We show that the CNN approach delivers species compositions comparable to those from OBITools and historical records. Fish assemblages retrieved using OBITools and CNN were consistent with the current knowledge on Guianese fish fauna, with marked differences between coastal and inland sites^29^. Fish homogeneity in coastal areas was explained by a historical connectivity between the coastal basins during the Miocene^21^, but also by the salt tolerance of a substantial number of the fishes inhabiting coastal streams^45^. Composition analysis further highlighted sites with a markedly different fauna, corresponding to the areas heavily disturbed by gold mining, forestry and agriculture^4^. In only a few minutes, the software transformed a raw fastq sequence data set into a species list associated with each eDNA sample collected in the field, which can serve further biodiversity analyses. Overall, our findings indicate that machine learning offers new possibilities for the taxonomic labelling of short DNA sequences and can transform rapidly collected eDNA data samples into interpretable taxonomy-based biodiversity indicators^25,30,68^.

In classical bioinformatic pipelines, the processing from raw sequence reads to taxonomic identifications includes seven steps (paired-end read merging, demultiplexing, dereplication, quality filtering, removal and correction of PCR/sequencing errors, and taxonomic labelling) expected to be essential to generate high-quality results from metabarcoding studies, but which can be computationally demanding^8,16^ and challenging to articulate^50^. We show that a CNN can embed all these steps in a single process applied directly to the raw Illumina reads when the CNN is trained to handle noisy data. Moreover, for relatively short eDNA markers (e.g. 60 bp for the ‘teleo’ marker used here), merging paired-end reads is not necessary, which leads to a significant computational gain^52^. While still offering results roughly comparable to those of OBITools, the CNN decreases the processing time of the whole data set analysis by a factor of around 150. In a recent comparison, Barque (https://github.com/enormandeau/barque) combined with a fast demultiplexing module was able to process over 15 million reads in 30 min, while it took 17 h for OBITools V1^52^. Assuming the same rate as found for our CNN, i.e.  1 million read per minute, to this data set, the application of the CNN would be two times faster than the fastest existing bioinformatic pipeline in a single model^52^. Our study represents a first successful adaption of CNN to the processing of eDNA metabarcoding data, but we foresee several avenues of optimization to gain speed and accuracy, making it a promising tool for scaling-up biodiversity inventories via eDNA^5,64^.

The training of CNNs leads to an efficient adjustment to the reference database, avoiding the need to explore a large number of parameters and arbitrary thresholds, as required in classical bioinformatic pipelines. Existing bioinformatic pipelines contain a variety of modules (i.e. QIIME2, DADA2, Vsearch), each with its own set of parameters^7,17,62^. Selecting the appropriate modules and parameters requires advanced knowledge of the functioning of the program, since changes in those parameters can considerably modify the outputs^8,13,36^. The absence of an appropriate and automated method for parameter optimization^2^ often limits the use of those pipelines by non-specialists. In contrast, the application of a CNN only includes a first step of training, where the optimization of the network is nearly automated, and two independent steps for applying the CNN and demultiplexing the reads to reach to final taxonomic outputs per sample. During the learning step of a CNN, only three parameters have to be set by the user: the network size (number of layers, filters and units), the learning rate, and the augmentation values. During the application step, two parameters are optimized, the binarization threshold and the minimum number of reads per sample to be considered. We expect that these steps can be nearly automated within a user-friendly software, as developed for other machine learning applications^76^. Given the relative ease of the training process and application of CNN, the approach could be transformed into an application with a user-friendly interface demanding only a minimum amount of interaction. Hence, CNNs could make eDNA metabarcoding data processing accessible even to less trained users and provide an overview of biodiversity more rapidly.

A CNN trained on a complete reference database produced species composition outputs congruent with the outputs of a popular bioinformatic pipeline, but showed a tendency to predict more species than those of OBITools and historical records. Compositional differences in the outputs of pipelines have already been highlighted (e.g.^11^) and have mainly resulted from the detection of several false positives and false negatives^52^. With a binarization threshold of 0.9 optimized during the training phase, we found congruent but slightly divergent results between OBITools and the CNN applied to either the raw or clean reads. While the CNN and OBITools shared most of their recovered species, each method detected a few species not detected by the other approach (Fig. 4). However, the CNN showed a general tendency of overprediction compared with OBITools and the historical records, especially when it was applied directly to the raw sequencing data. Using the historical records as a baseline, the CNN applied to clean reads reduced the detection of species only found with the CNN, without decreasing the number of species shared with OBITools or historical records, suggesting false positives resulting from noisy inputs. Specifically, the CNN applied to raw reads detected more species from the Loricariidae, Cichlidae and Characidae families that were not found with OBITools, which may have been the result of sequencing errors that were not denoised by the CNN. In the case of the Cichlidae family, the short barcode we used is known to be poorly resolved^73^, with many species sharing the same sequence^61^, and our CNN did not perform well in this situation, like all other pipelines. Moreover, Loricaridae and Characidae are the two most speciose families of the Guianese fish fauna, with more than 50 species per family^45^ and with several new species occurrences recorded each year in Guianese rivers (e.g.^12^). These two families, together with Cichlidae, are also known to host cryptic and still unnamed species, as shown by Papa et al. for the Maroni river^57^. This could also contribute to species misdetections. Finally, we found that the correlation between OBITools and CNN was lower at the sample level than at the level of the PCR replicates when the CNN was applied to raw reads. Hence, appropriately combining the PCR replicates could confer more robustness to the final outputs of the CNN. Refinement of the network could be added, so that the detection across multiple PCR replicates could be used to compute the final likelihood.

In our study we proposed a novel application of a CNN approach to eDNA metabarcoding data, but several improvements are required before broad-scale applications to large eDNA data sets can be considered. The CNN trained in this study learns from the species class and is forced to assign the sequences to that taxonomic level. Thus, when presented with conflicting sequences, the network might assign all of them to a single species, or may split the probabilities across several species, which might then be discarded given the use of the 0.9 binarization threshold. In contrast, in the case of a conflict, OBITools can assign sequences to higher taxonomic levels, thus keeping information related to these species with identical sequences. In this case study, we had an ideal situation where the reference database was almost complete for the territory. The CNN could be improved to handle incomplete reference databases and to be able to assign a read to another taxonomic level or to an unknown class, rather that forcing a species-level identification and relying on the binarization threshold to reject unknown sequences. Further, we expect that it is possible to improve the CNN by implementing more stringent filters that would reduce the number of false detection and prediction errors. For instance, a filter for tag-jump handling, included in previous pipelines for eDNA metabarcoding for fish (e.g.^22^) could be considered. Finally, while the computational speed was already faster than existing traditional pipelines, specific optimizations, such as network pruning or lower precision computations, could improve the performance further, making this approach even more attractive for applications in future broad-scale eDNA projects.

### Conclusion and perspectives

We have demonstrated that we can use deep learning to increase the speed and decrease the energy consumption required for processing eDNA metabarcoding data, with a high accuracy when applied to clean reads and a slightly lower accuracy for raw reads. The largest part of the computation time for the CNN is for the training phase; once trained, the CNN can be used as a computationally efficient tool for applications in the cloud, facilitating analyses of the mass of eDNA data expected to be collected in future biodiversity surveys. eDNA data are being collected at an exponentially increasing rate. Owing to its easy application—due to the reduced number of processing steps and the automated learning of best-suited parameters, a CNN approach contrasts with other widely-used bioinformatic pipelines. Our work paves the way towards computationally efficient and user-friendly online processing pipelines that will contribute to the democratization of bioinformatic analyses of eDNA samples. Our work is a major complement to the recent development and standardization of eDNA in the laboratory; together, they will make it possible to extend the use of eDNA in community ecology and biogeography, even for poorly understood ecosystems or lineages^43^, and they will help to install eDNA as a standard monitoring tool^42^. Our findings also reinforce the initial goal of quick and efficient eDNA application for biodiversity monitoring. We expect that the results from this study will be scaled up to help CNNs become a major toolkit for ecological analyses of eDNA data, possibly associated with a cloud infrastructure and parallel computation on GPUs.

## Material and methods

### eDNA data collection and reference database

As a test data set we used data collected in French Guiana, a *c*. 80,000 $km^{2}$ South American territory almost entirely covered by dense primary forest (Supplementary Material Fig. 2). The equatorial climate, associated with abundant rainfall, has created a dense hydrographic network consisting of six major watersheds and several coastal rivers that host a highly diverse fish fauna with at least 368 strictly freshwater fish species^45^. eDNA field collection was initiated in 2014 and continued until 2020. We sampled over 200 sites (see Murienne et al.^54^ for details), where we filtered 30 litres of river water across a flow filtration capsule using a peristaltic pump. For the purposes of this study, we analysed only the filters collected in both the Maroni and Oyapock rivers.

At each site we collected one to ten filtration capsules, but at most sites two capsules were used (2 $\times$ 34 l), using a previously established protocol^20,26^. We used a peristaltic pump (Vampire sampler, Burlke, Germany) and disposable sterile tubing to pump the water through the encapsulated filtering cartridges (VigiDNA 0.45 $\upmu$M, SPYGEN, France). We held the input part of the tube a few centimetres below the surface in rapid hydromorphologic units to facilitate homogenization of DNA in the water column. When the filters began to clog, we decreased the pump speed to avoid material damage. To minimize DNA contamination, the operators remained downstream from the filtration site, either on the boat or on emerging rocks. After filtration, we filled the capsules with a preservation buffer and stored them in the dark at room temperature for less than 1 month before DNA extraction. We applified the 12S rRNA ‘teleo’ gene fragment^77^ using PCR and sequenced it on an Illumina platform, generating an average of 500,000 paired-end sequence reads per sample. The DNA extraction, amplification and sequencing protocol have been described previously^19^.

We generated an eDNA reference database by combining fish specimens caught using various types of fishing gear. These data were complemented by fish collections carried out by environmental management agencies (DGTM Guyane, Office de l’eau Guyane, Hydreco laboratory), fish hobbyists (Guyane Wild Fish), and Museum tissue collections (MHN Geneva). Although rare for Guianese fishes, we also included existing sequence data from online databases (Genbank, Mitofish). We extracted and sequenced the 12S ribosomal gene from the collected species. The local reference database has improved over the years^20,22^ and now covers over 368 species out of 380 estimated to occur in the region. This almost full coverage is exceptional considering the many gaps globally^51^. Sample collection was authorized by both the French Ministry of Environment (DEAL) and the Guyanese National Park (PAG). The samples comply with the international rules of the Nagoya protocol for access and benefit sharing (project refs ABSCH-IRCC-FR-246820-1 and ABSCH-IRCC-FR-245902-1).

### OBITools bioinformatic pipeline

As a standard processing pipeline we selected OBITools^10^, which is commonly used in eDNA metabarcoding studies^15,47,78^. We processed the reads from the sequencing following Valentini et al.^77^. In short, we assembled the forward and reverse reads using illuminapairedend with a minimum score of 40, retrieving only joined sequences. We then assigned the reads to each sample using ngsfilter. We then created a separate data set for each eDNA sample by splitting the original data set into several files using obisplit. After this step, we analysed each sample individually before generating the taxonomic list. We clustered strictly identical sequences together using obiuniq. Further, we excluded sequences shorter than 20 bp using obigrep. We then ran obiclean within each PCR product for clustering. We discarded all sequences labelled as ‘internal’, corresponding most likely to PCR substitutions and indel errors. We performed taxonomic labelling of the remaining sequences using ecotag with the custom genetic reference database relevant for the eDNA samples. Finally, we applied an empirical threshold to account for tag-jumps and spurious errors.

### Reference data augmentation and training data set

The reference database has a full species coverage, but the number of DNA replicate sequences for each species was limited because there were only 683 sequences for 368 species. This makes training a CNN challenging for several reasons. The number of sequences per species is not balanced, there are not enough sequences to capture the entire inter- and intraspecific variation, and the noise from the sequencing process is not accounted for. To balance the data set using data augmentation procedures, we oversampled the underrepresented species before training. To increase the sequence variation, we implemented an inline sequence mutation step similar to that applied by Busia et al.^14^. During each training epoch all sequences were randomly mutated. We added between zero and two random insertions and deletions each, as well as noise in the form of a 5% mutation rate. This procedure further reduced overfitting, as no training sample was likely to be repeated twice. For the evaluations, we either added no augmentation or 2% noise and singular insertions and deletions, as we expected the PCR amplification and sequencing to be better than the 5% noise considered during the training phase.

For the direct application on the raw reads, another data transformation step was required. All sequences processed in an Illumina machine retain the selected primers, and were tagged with 8-bp-long tags. During the sequencing two bases from the plate attachment sequence were often read as well. We therefore pre- and appended the forward and reverse primers, and the combined tags and attachment bps to the sequences from the reference database. Specifically, we added 10 bp of unknown bases to each reference sequence, represented by the IUPAC code ‘N’. This shifted the sequences to a position in the training input similar to where they would occur in the Illumina data. While there is a canonical read direction for DNA, the read direction during the sequencing randomly occurs on either DNA strand. Therefore, we added the reverse complement of all sequences to the final data set. As a last step we truncated all sequences to a read length of 150 bps, as fixed by the field metabarcoding data.

### Convolutional networks

CNNs play a key role in modern computer vision applications and date back to the emergence of artificial neural networks in the 1950s and 1960s. Some of the first applications of CNNs and their training method include digit recognition for handwritten ZIP codes^46^. Each convolutional layer in a CNN consists of a number of convolutional kernels often called filters. These filters can be thought of as feature detectors each responding to a specific feature in the input data. Compared with fully connected dense layers, the small extent of these filters drastically reduces the number of free parameters to train. Intuitive examples in image processing are edge or corner detectors. By arranging the DNA sequences as two-dimensional inputs, the convolutional layer can learn and exploit abstract features in the sequences.

### CNN training and evaluation using split sampling

We investigated the performance of a CNN approach trained on the reference database at the species level. To encode DNA sequence information, each canonical base (A, C, T, G) and each IUPAC ambiguity code was translated to an appropriate four-dimensional probability distribution over the four canonical bases (A, T, C, G), including uncertain base reads (e.g. W and S). For example ‘A’ became [1,0,0,0] and ‘W’ became [0.5, 0, 0, 0.5]. The neural network was designed and optimized through a series of tests that allow the optimal set of correct DNA features to be selected. In particular, we explored an exhaustive number of model sizes, including one to three layers of 2D (depth-wise separable) convolutions with 4–16 filters each, one to three fully-connected layers with varying numbers of neurons each, and a softmax activated output layer which produces a probability distribution over all possible taxonomic labels. We applied dropout regularization and used leaky rectified-linear activation for all but the last layers.

We used TensorFlow^1^ to train the CNNs with all the aforementioned data augmentations. Due to the sparse data set, we characterized and evaluated the performance of the neural networks using three different methods. First, we applied random split-sampling from the reference database. This established a proper separation between the training and validation data, but less than half the species in the reference data set had two or more sequences, resulting in a reduced range of species that could be included. Specifically, only 156 out of 368 species possessed two or more unique sequences and were considered for the split data set. Next, we trained several networks on the full reference data set with all 368 species and validated them using the original non-augmented reference data. We derived more synthetic data from the reference sequences similar to the training augmentations and evaluated them with the chosen network. We evaluated whether there were systematic errors in the CNN performance. We further investigated whether a binarization threshold, requiring the probability of the most likely prediction to be above a certain value, improved the classification performance. As we prioritized the absence of errors, i.e. fewer false positives, over the presence of correct predictions, we evaluated the effects of such a binarization threshold using the F-beta measure, which uses a weighted trade-off between these errors. We chose a small beta value of 0.3 to heavily discourage false positives at the cost of discarding some correct results.

### CNN application on demultiplexed and cleaned samples

We tested the best trained CNN on the curated eDNA reads after the application of the main cleaning steps of the OBITools pipeline. In particular, from the Illumina raw output, we assembled the forward and reverse reads using the illuminapairedend algorithm from the OBITools package, after which we kept only high-quality reads and demultiplexed them across the different eDNA samples. We applied the best-trained CNN at the species level to these curated eDNA samples. We compared the taxonomic labelling performed by the CNN to classic labelling using ecotag from OBITools. We evaluated and applied different thresholds for accepting species detection as a way to remove spurious errors and wrong assignments (0, 5, 10, 25, 50, 75 and 100 reads in at least one PCR replicate). For each eDNA PCR replicate and filter, and for the whole rivers, we ranked the taxonomic groups by the number of reads recovered by each method and performed a Kendall rank correlation. We ran one rank correlation per eDNA sample and reported the median rank correlation across all samples. In addition, we compared the presence–absence using the kappa statistic, which measured the general agreement between the methods for each sample. We calculated the median percentages and median kappa values across the samples. Then, across all eDNA samples, we correlated the species richness obtained via CNN with that obtained with OBITools. Each analysis was performed at three different scales: the PCR replicate, the filtration capsule and the river. Finally, we ordinated the species composition of each filtration capsule for both methods using a principal coordinate analysis (PCoA), to compare differences in recovered compositions among the methods.

### CNN application on the raw illumina sequences

We applied the best CNN directly to the raw outputs from the Illumina sequencing, where we omitted all the preprocessing steps from OBITools. The CNN was expected to learn how to ignore the primer, as it was constant for all presented sequences. Furthermore, the output sequences from the Illumina sequencer were fixed in length (150 bp), so we fixed the input width of the CNN to this size. We systematically zero-padded or truncated the input sequences to this length during training, evaluation, and application. After the training phase with the reference database and the application on fastq, we developed a custom code for the fast demultiplexing of the reads. By focusing on the tag information in the first few positions of the sequence and not considering read errors in tags, we reduced the demultiplexing to a few simple look-ups in a hash table (currently 5), therefore reducing computation time with limited information loss. As in the previous test, we obtained a list of taxonomic labels for each eDNA sample, which could be compared with species composition information obtained with the OBITools pipeline. We further applied a threshold approach, obtaining a predicted composition per sample for any threshold tested. As done previously, for each eDNA sample, we ranked the taxonomic groups by the number of reads recovered with each method and performed a rank correlation. We calculated the median rank correlations across all the eDNA samples. In addition, we compared the presence–absence at the species level using the overall kappa statistic. We further evaluated whether differences between methods were more frequent in specific taxonomic families than others. Then, across all eDNA samples, we correlated the species richness obtained via CNN with that obtained with OBITools, and ordinated species composition of each filter for both methods on a PCoA. We evaluated the change in accuracy between the CNN applied to curated reads compared with the CNN applied to raw fastq files.

### Validation with existing biodiversity knowledge on the region

We compared the species composition recovered in the eDNA samples by CNN and OBITools to the species, genus and family checklists of each river catchment. Species lists for each catchment were obtained from an updated version of the catchment-scale species lists^45^ provided in Le Bail et al. From this list, we updated the taxonomy and added novel occurrences of known species based on fish catches by several research and management organizations (see ‘Material and methods’ section). Only collected specimens with a validated taxonomy were considered when updating this list, and detections using eDNA were not considered. We specifically quantified the number of matching species, false presences and false absences from each method, taking the checklists as references.

## Supplementary Information

Supplementary Information 1.

Supplementary Information 2.

## Data Availability

Partial data is available through Cilleros et al.^22^. The full data set is available from the corresponding author upon request.

## Code availability

The code is available in the supplementary material and released under the AGPLv3 license.
