# Supplementary material for: Applying convolutional neural networks to speed up environmental DNA annotation in a highly diverse ecosystem
Source: Sci Rep. 2022 Jun 17;12:10247. doi: 10.1038/s41598-022-13412-w (PMC9205931; doi:10.1038/s41598-022-13412-w)
Supplement: Supplementary file 1 — Supplementary Information 1. [file 41598_2022_13412_MOESM1_ESM.pdf]

Supplementary Material for: 'Applying convolutional neural networks to speed up environmental DNA annotation in a highly diverse ecosystem'

Benjamin Flück [1,2*], Laëtitia Mathon [3], Stéphanie Manel [3], Alice Valentini [4], Tony Dejean [4], Camille Albouy [5], David Mouillot [6,7], Wilfried Thuiller [8], Jérôme Murienne [9], Sébastien Brosse [9], and Loïc Pellissier [1,2*]

[1]Department of Environmental System Science, ETH Zürich, 8092 Zürich, Switzerland
[2]Swiss Federal Research Institute WSL, 8903 Birmensdorf, Switzerland
[3]CEFE, Univ. Montpellier, CNRS, EPHE-PSL University, IRD, Montpellier, France
[4]SPYGEN, Le Bourget-du-Lac, France
[5]IFREMER, unité Écologie et Modèles pour l'Halieutique, rue de l'Ile d'Yeu, BP21105, 44311 Nantes cedex 3, France
[6]MARBEC, Univ. Montpellier,CNRS, IRD, Ifremer, Montpellier, France
[7]Institut Universitaire de France, IUF, Paris 75231, France
[8]Univ. Grenoble Alpes, Univ. Savoie Mont Blanc, CNRS, LECA, Laboratoire d'Écologie Alpine F- 38000 Grenoble, France
[9]Laboratoire Evolution et Diversité Biologique (UMR5174), CNRS, IRD, Université Paul Sabatier, Toulouse, France
[*]Corresponding authors: [benjamin.flueck, loic.pellissier]@usys.ethz.ch

May 24, 2022

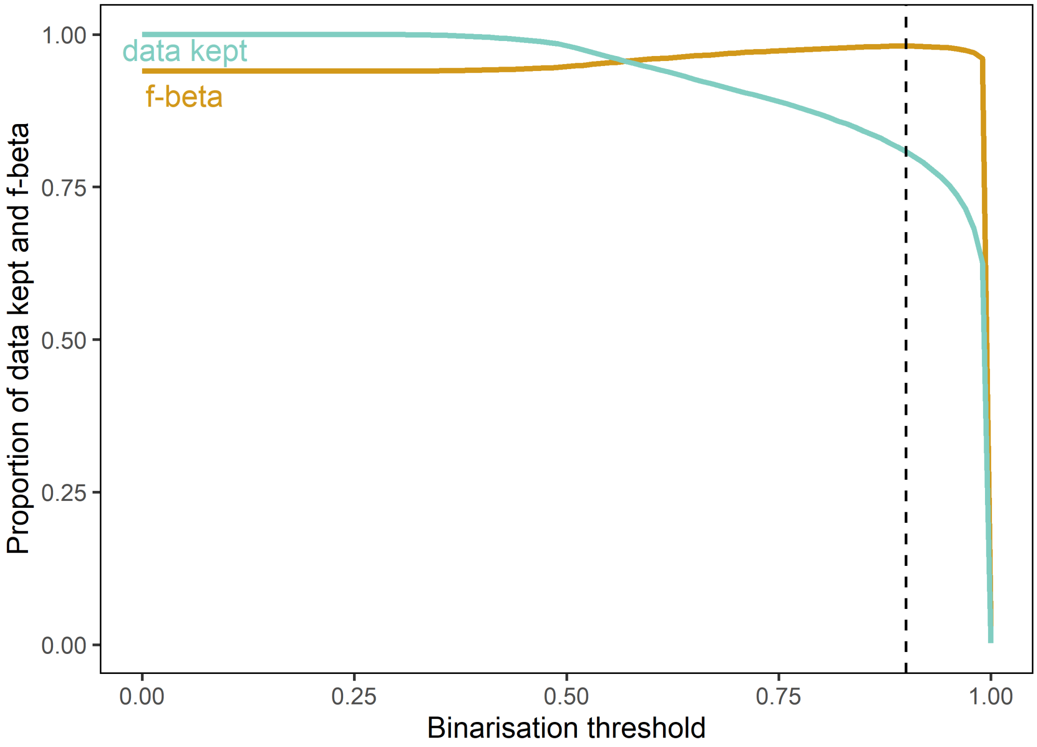

Figure 1: F-beta measure (orange line) based on the predictions of the CNN on synthetic data after the training phase, for each binarization threshold value. The proportion of data discarded for each binarization threshold is also shown (blue line). The dashed vertical line indicates the threshold of 0.9, providing the highest F-beta value with the minimum amount of data discarded.

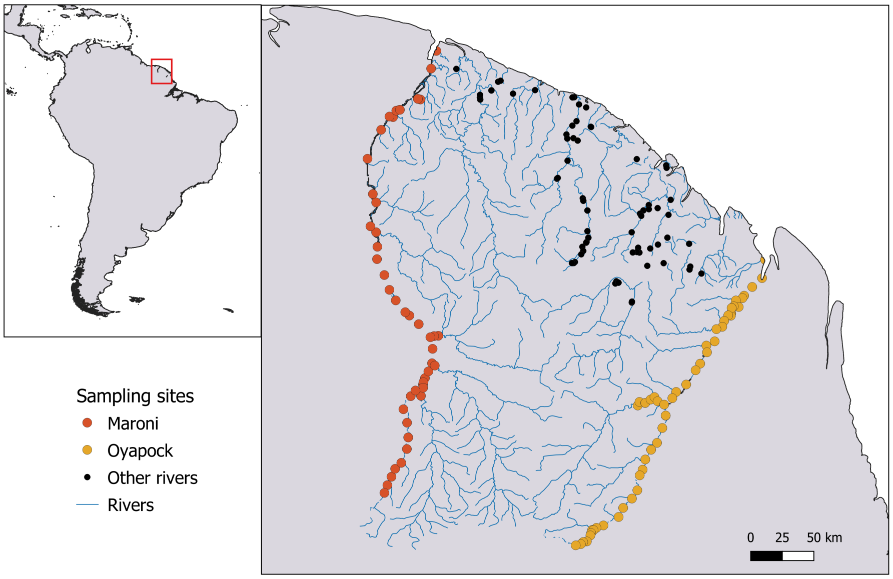

Figure 2: Map of the sampling locations in French Guiana. Sampling sites were located on the Maroni river (red), on the Oyapock river (orange), and on other rivers (black). The maps were created with QGIS 3.6.1

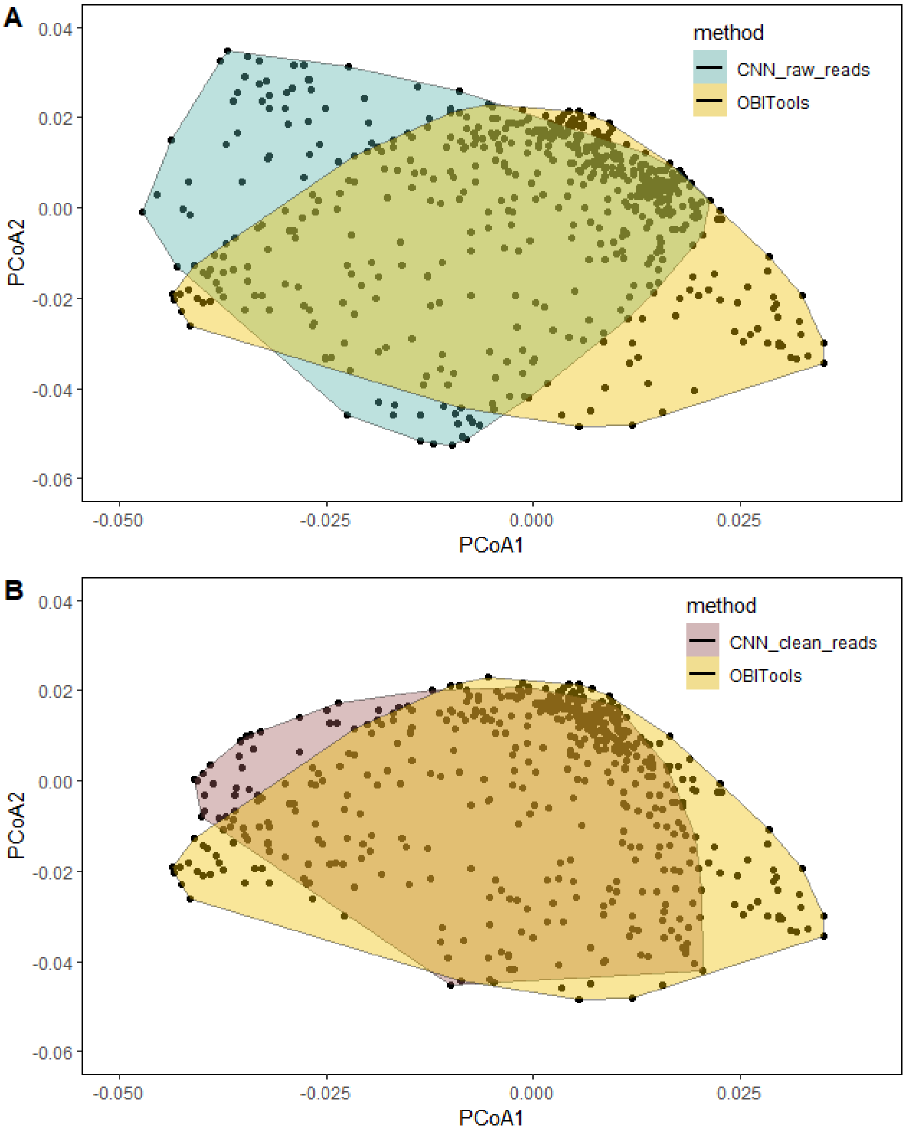

Figure 3: Principal coordinate analysis (PCoA) of species composition similarity between filters. A: Ordination of filter species composition similarity in the outputs of the CNN applied to raw reads (blue) and in the outputs of OBITools (yellow). B: Ordination of filter species composition similarity in the outputs of the CNN applied to clean reads (red) and in the outputs of OBITools (yellow). Similarity matrices were built with Bray-Curtis distances on read abundance per species per filter.

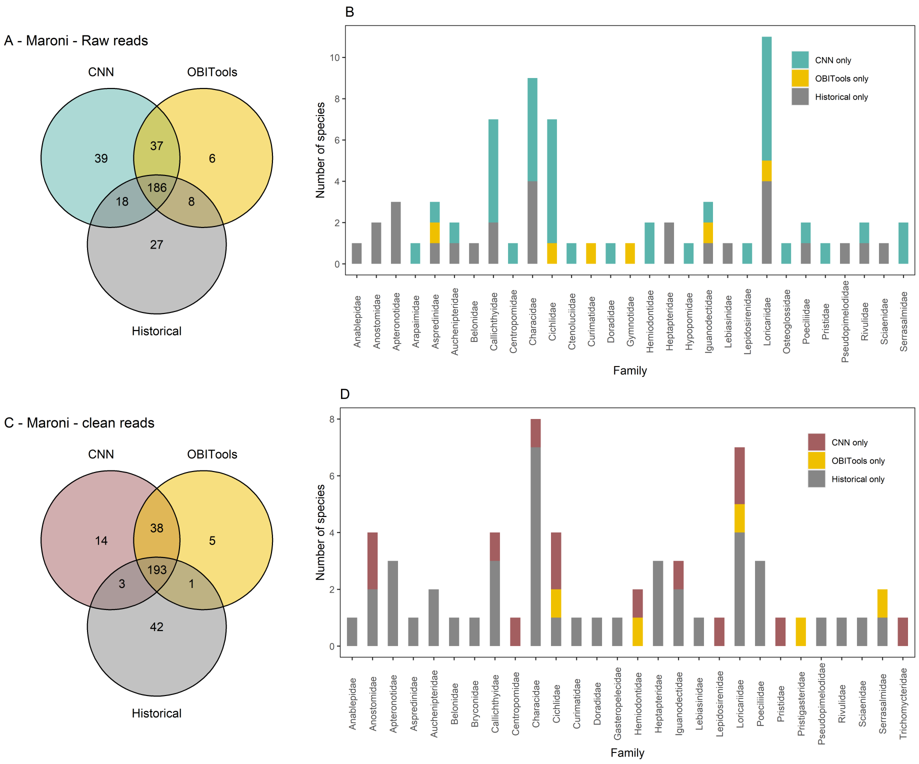

Figure 4: Species detections with the CNN, with OBITools and in historical records in the Maroni river. A: Overlap of species detections between the CNN applied to raw reads (blue), OBITools (yellow) and historical records (grey). B: Number of species per family detected with only one method (CNN applied to raw reads, OBITools or historical records). C: Overlap of species detections between the CNN applied to clean reads (red), OBITools (yellow) and historical records (grey). D: Number of species per family detected with only one method (CNN applied to clean reads, OBITools or historical records).

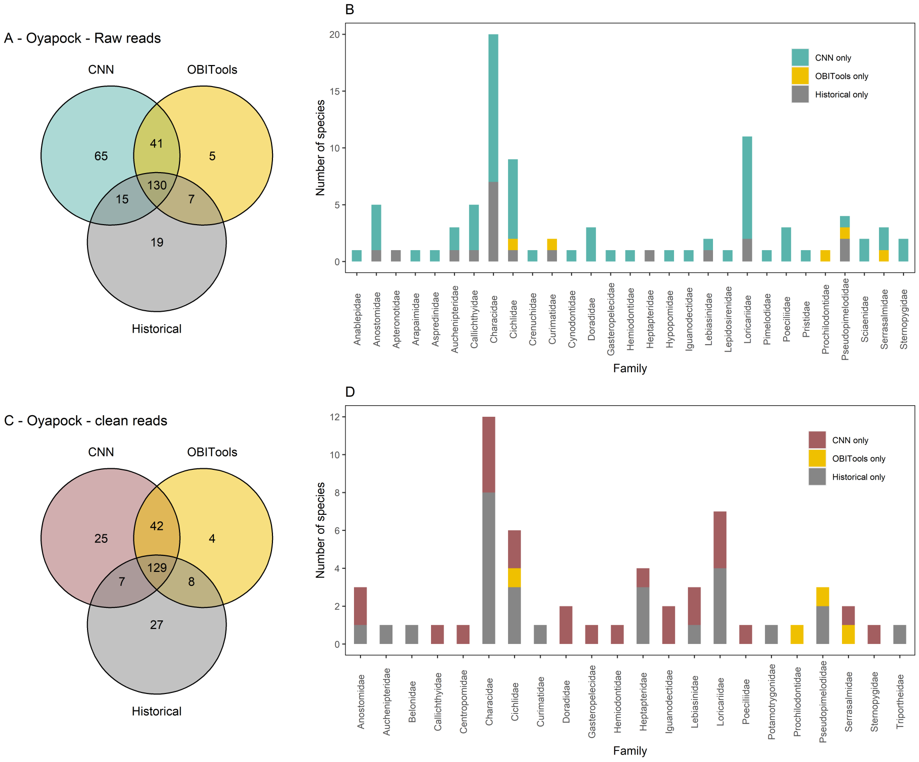

Figure 5: Species detections with the CNN, with OBITools and in historical records in the Oyapock river. A: Overlap of species detections between the CNN applied to raw reads (blue), OBITools (yellow) and historical records (grey). B: Number of species per family detected with only one method (CNN applied to raw reads, OBITools or historical records). C: Overlap of species detections between the CNN applied to clean reads (red), OBITools (yellow) and historical records (grey). D: Number of species per family detected with only one method (CNN applied to clean reads, OBITools or historical records).

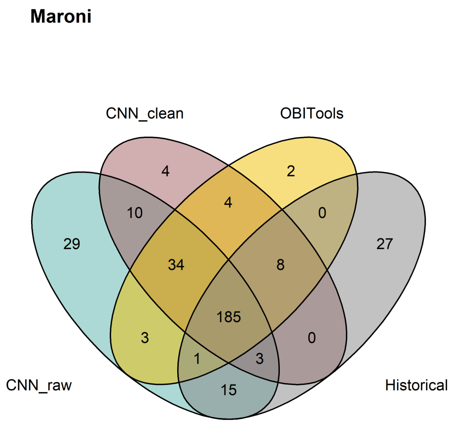

Figure 6: Species detections with the CNN, with OBITools and in historical records. Overlap of species detections between the CNN applied to raw reads (blue), the CNN applied to clean reads (red), OBITools (yellow) and historical records (grey) for the Maroni river (left) and the Oyapock river (right).
